# Plastome phylogeny and early diversification of Brassicaceae

**DOI:** 10.1186/s12864-017-3555-3

**Published:** 2017-02-16

**Authors:** Xinyi Guo, Jianquan Liu, Guoqian Hao, Lei Zhang, Kangshan Mao, Xiaojuan Wang, Dan Zhang, Tao Ma, Quanjun Hu, Ihsan A. Al-Shehbaz, Marcus A. Koch

**Affiliations:** 1 MOE Key Laboratory of Bio-Resources and Eco-Environment, College of Life Sciences, Sichuan University, 610065 Chengdu, People’s Republic of China; 2 Biodiversity Institute of Mount Emei, Mount Emei Scenic Area Management Committee, 614200 Leshan, Sichuan People’s Republic of China; 3 Missouri Botanical Garden, PO Box 299, St. Louis, MO 63166-0299 USA; 4 Department of Biodiversity and Plant Systematics, Im Neuenheimer Feld 345, Centre for Organismal Studies (COS) Heidelberg, Heidelberg University, 69120 Heidelberg, Germany

**Keywords:** Plastome, Brassicaceae, Phylogenomics, Molecular dating, Gene loss

## Abstract

**Background:**

The family Brassicaceae encompasses diverse species, many of which have high scientific and economic importance. Early diversifications and phylogenetic relationships between major lineages or clades remain unclear. Here we re-investigate Brassicaceae phylogeny with complete plastomes from 51 species representing all four lineages or 5 of 6 major clades (A, B, C, E and F) as identified in earlier studies.

**Results:**

Bayesian and maximum likelihood phylogenetic analyses using a partitioned supermatrix of 77 protein coding genes resulted in nearly identical tree topologies exemplified by highly supported relationships between clades. All four lineages were well identified and interrelationships between them were resolved. The previously defined Clade C was found to be paraphyletic (the genus *Megadenia* formed a separate lineage), while the remaining clades were monophyletic. Clade E (lineage III) was sister to clades B + C rather than to all core Brassicaceae (clades A + B + C or lineages I + II), as suggested by a previous transcriptome study. Molecular dating based on plastome phylogeny supported the origin of major lineages or clades between late Oligocene and early Miocene, and the following radiative diversification across the family took place within a short timescale. In addition, gene losses in the plastomes occurred multiple times during the evolutionary diversification of the family.

**Conclusions:**

Plastome phylogeny illustrates the early diversification of cruciferous species. This phylogeny will facilitate our further understanding of evolution and adaptation of numerous species in the model family Brassicaceae.

**Electronic supplementary material:**

The online version of this article (doi:10.1186/s12864-017-3555-3) contains supplementary material, which is available to authorized users.

## Background

The predominantly herbaceous family Brassicaceae (Cruciferae), which has some 3700 species, includes many vegetable crops in the genera *Brassica* and *Raphanu*s, sources of spices (*Eutrema* and *Armoracia*) and vegetable oils (*Brassica*), ornamentals (*Arabis*, *Hesperis*, *Lobularia*, and *Matthiola*), and model species in experimental biology (e.g., *Arabidopsis thaliana*). A robust phylogeny is crucial for diverse comparative studies. However, resolving the deep phylogeny of the family has been particularly challenging because its early evolution was extremely rapid [1–5], accompanied with ancient gene flow [6], polyploidization [7–9], and origin of novel traits [10]. Prior phylogenetic studies, which involved 325 genera and 51 tribes using sequence variations of a few chloroplast DNAs or ITS, identified four major lineages, with the basal lineage (tribe Aethionemeae) sister to the remaining three lineages (I, II, and III, i.e., core Brassicaceae) [2–5, 11–16]. The relationships between lineages within core Brassicaceae remained unsolved or inconsistent in those studies. Most recently, six clades were proposed based on phylogenetic analyses of low-copy nuclear genes retrieved from transcriptomes of 55 species [17]. The study further divided lineage II into three clades (B, C, D), but the remaining three clades were similar to the previously recognized three lineages (basal lineage, and lineages I and III). The clade E (lineage III) was sister to the remaining core Brassicaceae species (clades A + B + C or lineages I + II), but the relationship within the core were unsolved in the previous study [5]. Phylogenetic conflicts between different datasets, especially between nuclear and cytoplasmic genomes in plants, were found [18, 19], possibly suggesting complex evolutionary history.

The chloroplast genomes (plastomes), with extremely more informative sites for phylogenetic analyses than only a few DNA fragments, have proven to be highly effective in resolving disputed interrelationships in numerous plant groups [20–22]. Plastomes vary in size between 75 and 250 kb, have numerous copies in a given cell, inherited maternally in most plants, and have conserved gene content and order [23, 24]. The plastome is characterized by two usually large inverted repeat regions (IRa and IRb) separated by two single-copy regions referred to as the large single-copy region (LSC) and small single-copy region (SSC) that vary in length. Occasional structural changes, such as gene or intron losses, inversions, and rearrangements, were revealed by comparative genomic studies between groups. For examples, numerous plastome genes were lost multiple times in parasitic, nonphotosynthetic plants such as species of *Cuscuta* [25, 26], *Epifagus* [27], and *Rhizanthella* [28]. In photosynthetic species, the loss of chloroplast genes rarely occurs and only when the nuclear and/or mitochondrial genomes contain another functional copy or acquired one from the plastome through gene transfer [29]. Such rare cases were found in the *rpl22* gene in Fagaceae and Passifloraceae [30], the *rpl32* gene in *Populus* [31], and the *infA* gene in Brassicaceae [32]. Therefore, the relative stability of plastomes in plants provide highly orthologous alignments of large genome data that are valuable for phylogenetic analyses and calibrated divergence estimation [33–35].

The first plastome phylogeny of Brassicaceae have recently been presented aiming to provide a reliable temporal evolutionary framework within the entire clade of Superrosidae angiosperms and using critically evaluated fossil data for calibrating divergence-time estimates [35]. This was urgently needed because of conflicting hypotheses on Brassicaceae divergence-time estimates [36]. We built on this study [35] and expanded our sampling to 51 Brassicaceae plastomes and *Cleome* as outgroup. These species cover all four lineages or 5 out of 6 clades identified before [5, 17]. We aimed to examine whether the plastome dataset could: (1) significantly support the previously shown deep splits; (2) resolve the disputed interrelationships between lineages or clades; and (3) reveal any previously overlooked structural evolution within Brassicaceae plastomes.

## Results

### Basic characteristics of Brassicaceae chloroplast genomes

The average length of the plastomes from 53 species of Brassicales (Additional file 1: Table S1) is 154 kb, ranging from 152,659 bp in *Lobularia maritima* to 160,100 bp in *Carica papaya* (Additional file 1: Table S2). The average GC content is 36.4, 34.1, 29.3 and 42.3% for complete sequences, LSC, SSC and IR regions a and b, respectively, and varies slightly between species (Additional file 1: Table S2). As in the vast majority of angiosperms, both gene content and gene order are highly conserved, where the typical quadripartite organization harbored 132 genes including 79 protein coding genes (PCGs, 8 duplicated in IR), 30 tRNA genes (7 duplicated in IR) and 4 rRNA genes (4 duplicated in IR).

### Sequence alignment and evaluation of data partitions

Based on the 77 PCGs, a gap-free supermatrix containing 64,962 sites was concatenated, of which 7611 were parsimony informative (Additional file 1: Table S3). The aligned lengths of these PCGs ranged from 84 to 6645 bp (mean = 844 bp). No significant compositional heterogeneity among sequences was detected for any genes among species (Additional file 1: Table S4). The combined 77-gene data set displayed no apparent substitution saturation (Additional file 1: Table S4). Evaluation of partition strategies suggested that the automatically determined scheme is the best according to Bayesian information criterion (BIC) and the most parameter-rich gene-codon model is generally better than the less partitioned ones, while the codon-partitioned model was favored over the gene-partitioned model (Additional file 1: Table S5).

### Plastome phylogeny

**Fig. 1:**
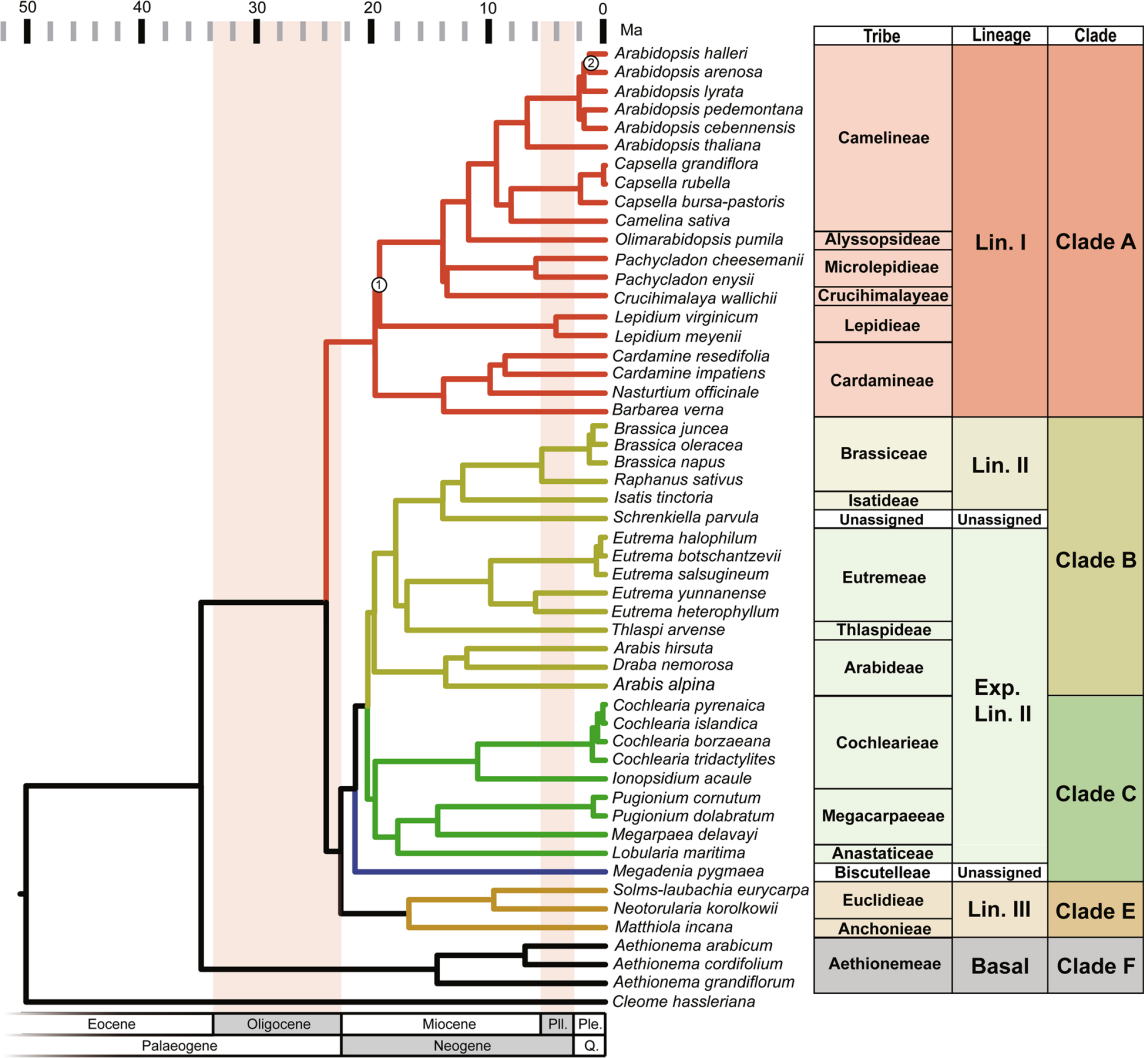
Phylogeny of Brassicaceae. Time-calibrated phylogeny of 51 Brassicaceae species inferred from a concatenated Bayesian analysis of 77 plastome protein-coding genes with all mutations using BEAST2. Higher taxon names appear at *right*. All nodes are supported with posterior probability (PP) of 1.0, except for two marked with *circles* (Node1: PP = 0.558; Node2: PP = 0.833). Geological periods were marked with background colors. *Ma* million years ago, *Ple* Pleistocene, *Pli* Pliocene, *Q.* Quaternary

Both RAxML and BEAST analyses of the concatenated sequence supermatrix produced similar topologies for the 53 species. For ML analysis, the topology and support values for specific splits varied using different partition schemes or subsets. After a visual check, no well-supported conflicts (i.e., those receiving bootstrap (BS) >90%) were found between individual genes trees. Regardless of the data partition strategy in our ML analysis, the majority of relationships across the family were consistent and well supported. The BEAST topology based on the best-partition scheme defined by partition finder produced well-resolved phylogeny for all but two nodes (Fig. 1). In line with a recent conclusion [17], the three previously proposed lineages are placed into different clades. The placement of tribe Lepidieae was unstable across the analysis, and the alternative topology could not be rejected by approximately unbiased (AU) test (*P* = 0.297, Table 1). However, the relationship between clades determined by plastomes is discordant with nuclear gene phylogeny [17]. Of particular interest is the recently defined Clade E, a lineage containing a majority of Lineage III species, which is sister to the combined BC clade. Thus, after the split with basal Aethionemeae species, the core Brassicaceae diverged into two large clades. The first clade included species from Lineage I (or Clade A), while the second clade consisted of species from all other major lineages or clades except for the newly identified Clade D because of the limited taxon sampling. In addition, we found that *Megadenia*, a genus placed in the tribe Biscutelleae of Clade C, is sister to all other species of Clades B and C. Multiple tests confirmed the relationship recovered here and rejected the alternative phylogeny as previously proposed [17] (*P* < 0.01, Table 1).

**Table 1:** Comparison of tree topology hypotheses by using likelihood

Hypothesis	△lnL	AU	BP	PP	KH	WKH	SH	WSH
H1	Best	**0.756**	**0.721**	**0.977**	**0.73**	**0.73**	**0.966**	**0.986**
H2	3.7	**0.297**	**0.273**	0.023	**0.27**	**0.27**	**0.693**	**0.555**
H3	25.3	0.011	0.006	1.00E-11	0.014	0.014	**0.176**	0.026
H4	27.9	0.003	4.00E-05	7.00E-13	0.007	0.007	**0.145**	0.022
H5	118.1	8.00E-65	0	5.00E-52	0	0	0	0

Note: Tree Hypothesis: H1. This study; H2. (Other CladeA + Cardamineae) + Lepidieae; H3. (((A, E), (B, C)), F); H4. (((A, (B, C)), E), F); H5. *Megadenia* within Clade C. △lnL: the observed log-likelihood difference. *AU* approximately unbiased test, *BP* bootstrap probability test, *PP* approximate Bayesian posterior probability, *KH* Kishino-Hasegawa test, *WKH* weighted Kishino-Hasegawa test, *SH* Shimodaira-Hasegawa test, *WSH* weighted Shimodaira-Hasegawa test. *P* values >0.05 are in bold. The topology for each alternative hypothesis is provided in Additional file 2: Figure S1

### Fossil calibration and molecular dating

We included plastomes of 75 outgroups in order to allow the use of 14 non-Brassicales calibrations (Additional file 2: Figure S2; Additional file 1: Table S6). A clock rate partition test found two partitions for the whole alignment as the best fit scheme under relaxed lognormal clock model. Overall, the calculation of divergence times were barely affected by whether fossil calibrations within the Brassicales were used (Table 2; Additional file 1: Table S7). Also, there was no effect on age estimation whether we included the questionable *Thlaspi primaevum* fossil [37] (here used as a conservative constrain to the Brassicaceae crown node as sugg﻿ested ﻿[36]) or used the newly identified *Palaeocleome lakensis* fossil in the analysis [33] (Additional file 1: Table S7). According to the MCMCTREE time estimates, the core Brassicaceae and Aethionemeae began to split at 35.2 (30.0–42.5) Mya during the Eocene-Oligocene boundary (Fig. 1) while the origins of the major lineages or clades occurred between the late Oligocene and early Miocene (Table 2). These time estimates are broadly consistent with recent studies using large-scale genomic data [5, 17, 34, 35]. Remarkably, all major lineages or clades radiated within a short timescales window (~3 Myr between 17 and 20 Mya in the crown age; Fig. 1 and Table 2).

**Table 2:** Mean and 95% HPD Age Estimates from MCMCTree Analysis

Node	Brassicales Fossils	
	Used	Not Used
Cleomaceae vs Brassicaceae	44.5 - **50.5** - 59.1	39.5 - **49.0** - 57.6
Crown Brassicaceae	30.0 - **35.2** - 42.5	29.0 - **34.9** - 41.8
Crown core-Brassicaceae	21.7 - **25.3** - 29.7	21.3 - **25.1** - 29.8
Crown Clade A	16.9 - **20.3** - 24.2	16.5 - **20.0** - 24.1
Crown Arabidopsis	4.8 - **7.1** - 9.8	4.8 - **7.0** - 9.7
Crown Camelieae	7.5 - **9.9** - 12.8	7.4 - **9.7** - 12.5
Crown Cardamineae	10.2 - **14.2** - 18.6	10.0 - **14.0** - 18.3
Crown Clade B	17.6 - **20.6** - 24.5	17.2 - **20.3** - 24.4
*Brassica* vs *Schrenkiella*	11.4 - **14.7** - 18.3	11.2 - **14.5** - 18.2
Crown Eutremeae	6.5 - **10.1** - 14.3	7.4 - **10.0** - 14.2
Crown Arabideae	11.0 - **14.6** - 18.6	10.8 - **14.4** - 18.5
Crown Clade C	16.9 - **20.1** - 24.0	16.6 - **19.8** - 24.0
Crown Cochlearieae	7.8 - **11.6** - 15.9	7.6 - **11.5** - 15.9
Crown Megacarpaeeae	10.1 - **14.8** - 19.6	10.0 - **14.6** - 19.3
*Megadenia* vs BC clades	19.3 - **22.6** - 26.7	18.3 - **22.3** - 26.7
Crown Clade E	12.7 - **17.3** - 22.0	12.4 - **17.0** - 21.8
Crown Clade F	9.5 - **14.6** - 20.7	9.3 - **14.3** - 20.3

The numbers in boldface are mean values

### Gene loss across Brassicaceae

**Fig. 2:**
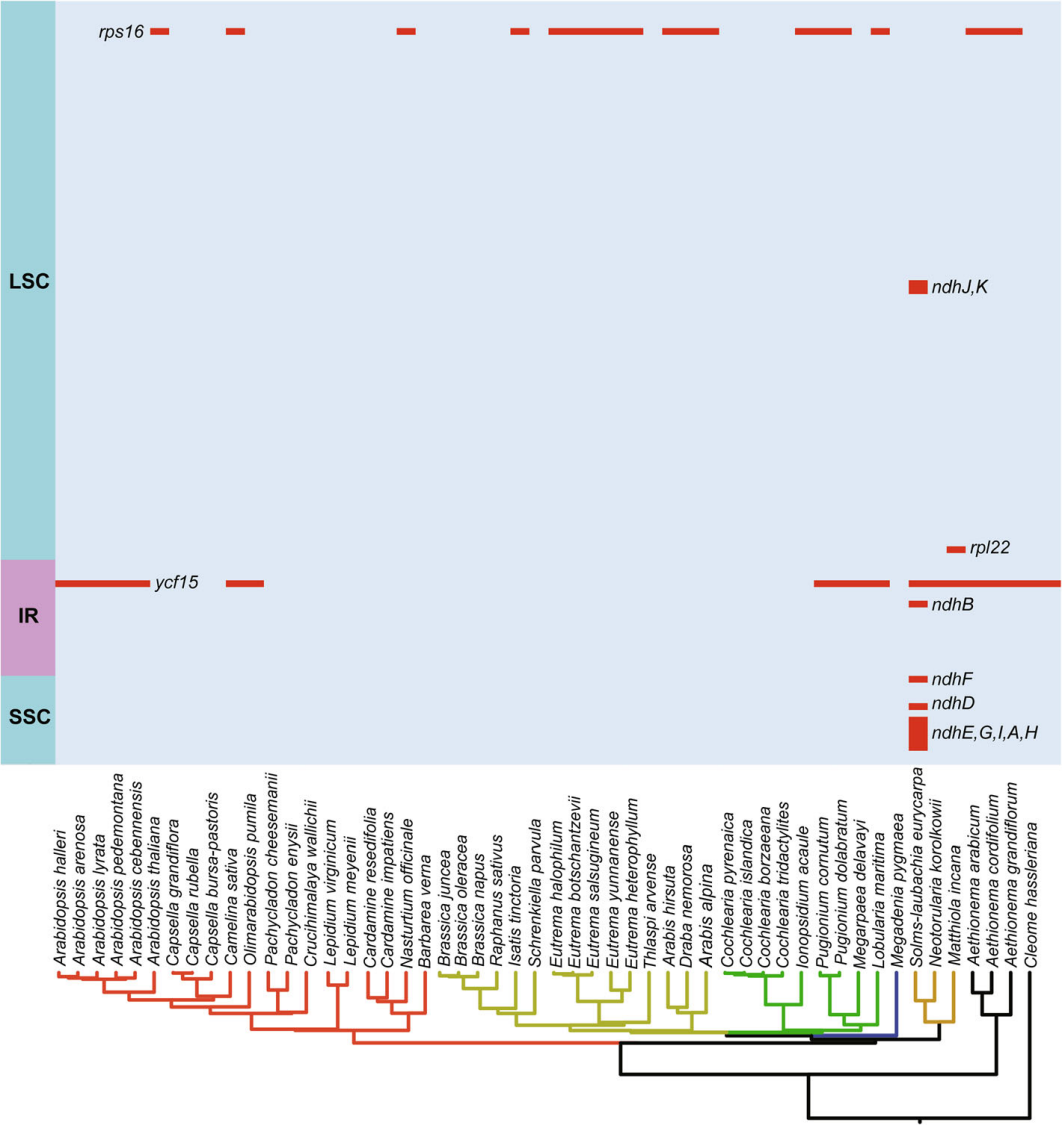
Loss of chloroplast protein-coding genes across Brassicaceae. Below are the phylogeny of Brassicaceae species based on chloroplast genomes as shown in Fig. 1. Different chloroplast regions were indicated at the *left side. Grey* and *red boxes* indicate intact and possible pseudogenized of genes, respectively. *IR* inverted repeat, *LSC* large single-copy region, *SSC* small single-copy region

As shown in Fig. 2, of the total 79 PCGs, 77 were predicted to be functional genes while *rps16* and *ycf15* became pseudogenes in some species (see also Additional file 2: Figures S3 and S4). Besides, the *rpl22* gene was slightly truncated in *Matthiola incana*. The only exception was found for *Solms-laubachia eurycarpa*, where 10 of the 11 *ndh* genes were either slightly or severely truncated due to premature stop codons.

## Discussion

In order to generate a backbone plastome phylogeny for Brassicaceae, we assembled 20 new plastomes to encompass all four lineages and 5 out of 6 major clades. All assumed lineages and major clades were generally identified, and their phylogenetic relationships were well resolved. In particular, our plastome phylogeny from 51 species provided the following new insights compared to those based on the fewer species plastome [35] or transcriptome genes [17]. First, Clade E, a group of Lineage III, is sister only to Clades B + C instead of sister to Clades B + C + A [17]. Clade A diverged from the group comprised Clades B + C + E very early. Second, the genus *Megadenia* of the tribe Biscutelleae in Clade C is sister to the remaining examined species of Clades B and C. This genus was shown to be closely related to *Biscutella* within the tribe Biscutelleae [38–41]. Thus, the phylogenetic relationship of the genus needs further examinations when more genera are sampled. Third, our comparisons based on different datasets suggested that the saturation in the third codon and phylogenetic signals from distinct plastome regions seriously affected the divergence and statistical supports in some particular nodes. For example, the tribes Lepidieae and others of Lineage I (Clade A) showed no distinct bifurcating divergence if all mutations of the total plastome dataset were used (Fig. 1). However, when excluding the third codon or using only slow-evolving IR genes, the Lepidieae diverged from Cardamineae and the others of the Lineage I (Clade A) with high statistical support (Additional file 2: Figures S5 and S6). *Pachycladon* was suggested to be closely related to *Crucihimalaya* [37, 42] as confirmed here by all plastome mutations (Fig. 1). However, this sister relationship was not supported when the IR gene dataset was used alone (Additional file 2: Figure S6).

Taxon sampling and reliable fossils used for calibrations are extremely important to estimate the divergence of targeted phylogeny [43–45]. Due to the high conservation and stable alignments, we used 14 highly reliable fossils from other eudicot orders [46] and three Brassicales. Our calibration comparisons suggested that the calculated divergences were rarely affected by Brassicales fossils, including the debated Brassicaceae fossil [37]. The estimated divergence times for major nodes were largely compatible with previous studies [5, 17, 34, 35], and it highlighted several evolutionary events. First, the stem age of Brassicaceae is around 50.5 Mya, ~6 Mya older than estimated by Magallón et al. [46] and ~5 Mya younger than estimated by Huang et al. [17], but consistent with a recent study across the Brassicales order [33]. Second, we confirmed that Brassicaceae began to diversify ~35.2 Mya during the Eocene-Oligocene boundary [35], when a warm and humid climate dominated the world [47]. Third, all major clades or lineages radiated within a short timescale between ~20 and ~17 Mya. All of these estimates are non-significantly different from those based on the plastomes with fewer Brassicaceae species [35], but younger than those based on the low-copy nuclear genes [17] with more representatives at the genus level. Therefore, both taxon sampling and evolutionary rates of different genomes might have caused differences in the estimated node times between different datasets.

A few chloroplast genes were lost in photosynthetic plants [29]. In this study, we reaffirmed the loss of the *rps16* gene in the LSC region [48] and found that the *ycf15* in the IR region became a pseudogene independently in different tribes of this family. Both genes were previously lost in other plants species [29]. The validity of *ycf15* as a protein-coding gene remains debated [49–51], and it may have a regulatory function [52] after the full transcription of the chloroplast genome [53]. Until now, the mechanism underlying the loss of the plastome gene in Brassicaceae has been poorly understood. The dominance of self-compatibility in the family might be related with the transfer and/or loss of some organelle genes [48, 54]. However, it should be noted that *Solms-laubachia eurycarpa* has lost most *ndh* genes. To our knowledge, this is the first report of the massive loss of the *ndh* genes in Brasssicales. A typical plastid genome contains 11 *ndh* genes that are highly conserved across most autotrophic seed plants, which indicates the presence of strong selection pressure for their retention. A complete loss of the plastid *ndh* gene was only reported in conifers, Gnetales, and some epiphytic plants [55, 56]. Further studies are needed to examine whether specific factors were associated with the loss of the *ndh* genes in the genus *Solms-laubachia*.

## Conclusions﻿

Recent eme﻿rgence of large scale phylogenomic data have undoubtedly provided a major advancement for understanding the complex systematics and taxonomy of the Brassicaceae, while phylogenetic relationships of the entire large family is far from being fully resolved. Using 51 chloroplast genomes from species of major cruciferous lineages or clades, we were able to resolve deep splits in this important plant family and found incongruence between organelle and nuclear genomes. The updated phylogenetic framework, based on plastome analysis, can be used to test many interesting evolutionary hypothesis on the origin and early diversification of Brassicaceae species. With the rapid increase in genomic data, we envision that a further in-depth understanding of the evolution of this model plant family will soon be possible.

## Methods

### Taxon sampling and plastome assembly

A total of 53 Brassicales species were included in this study, among which were 51 Brassicaceae species from 28 genera representing 19 out of the 51 tribes in all four major lineages or 5 out of the 6 newly identified clades. Plastome sequences were either obtained from the NCBI (last accessed, Jan 1st, 2016) or newly assembled (Additional file 1: Table S1). For the newly sequenced plastomes, we used the Illumina HiSeq X Ten sequencing pipeline to generate at least 2 Gb of 2 × 150 bp short reads data for each sample. Reads from the SRA database were extracted with fastq-dump software implemented in the SRA toolkit. We initially filtered reads following the previous approach [57]. Then, plastome contigs were assembled using Velvet [58], which were further reordered to the *Arabidopsis thaliana* plastome with SAMtools [59]. We finally merged all contigs into a consensus linear sequence using Geneious version 8.0.5 [60]. The annotation was performed with CpGAVAS [61] or Plann [62], aided by manually refinement in Apollo genome editor [63] and/or Sequin software [64]. Aragorn web-interface [65] was used to predict tRNAs.

### Sequence alignment and partition strategy

Protein coding genes (PCGs) were extracted from the Genbank formatted file containing all plastomes using customized Perl scripts, removing start and end codons. After excluding possible pseudogenes, a total of 77 PCGs were retained for all species except for *Solms-laubachia eurycarpa*, where the pseudogenized *ndh* genes were edited and included. Each PCG was aligned using PRANK v.130410 [66] according to the translated amino acid sequences. Ambiguous alignment regions were trimmed by using Gblocks 0.91b [67] with (−t = c) option to set sequence type to codons; otherwise the default settings were assumed.

To test the phylogenetic effects of different regions of the plastid genome, we created the following datasets based on different plastome partitions. All 77 refined PCG alignments were firstly combined into a concatenated data set and four different partitioning schemes: 1 partition (unpartitioned); 3 partitions (a separate partition for all first, second, and third codon positions); 77 partitions (one partition for each gene); and 231 partitions (a separate partition for the first and second codon positions together in each gene and a partition for the third codon position in each gene). In addition, a best-fit partitioning schemes and models were selected using the greedy search mode implemented in PartitionFinder v1.1.1 [68]. Comparisons of the five partitioning strategies and selections of corresponding nucleotide substitution models were conducted under the Bayesian information criterion (BIC). The best-fitting partitioning strategy found by PartitionFinder was used in the following analysis. In addition to the main dataset, we also extract subsets from the 77-gene alignments containing either first and second codon positions or third codon positions only to explore the effect of potential sequence saturation at third codon. The data matrices and resulting trees were deposited in TreeBase (http://purl.org/phylo/treebase/phylows/study/TB2:S20512).

### Phylogenetic analysis

The concatenated data set was first evaluated by BaCoCa [69], a recently developed program to perform multiple statistical analyses on multiple nucleotide and amino-acid sequence alignments, and then analyzed with Bayesian method and maximum likelihood (ML). The percentage of PI sites of each gene was estimated by PAUP [70]. The Bayesian MCMC analysis program BEAST (version 2.3.0) [71] was used to build phylogenetic trees, with parameter settings according to Hohmann et al. [35]. The GTR + G model was used for all ML analyses using RAxML version 8.0.20, as suggested in the manual [72]. Supports for nodes were assessed with 500 rapid bootstrapping replicates. Likelihood-based tests of alternative phylogenetic hypotheses were assessed based on the concatenated data set. Site-wise log-likelihoods of all alternative hypotheses (see Table 2) were first calculated with RAxML under the GTR + G model using the option (-f g). Then, the site log-likelihood file was supplied to the CONSEL v0.1j program [73] (Shimodaira and Hasegawa 2001) to estimate *P*-values for each alternative hypothesis using the AU test, approximate Bayesian posterior probability test, bootstrap probability test, Kishino-Hasegawa (KH) test, weighted KH test, Shimodaira-Hasegawa test (SH), and weighted SH test.

### Divergence-time estimation and fossil calibration

We used the latest MCMCTREE in the PAML4.9a package to estimate divergence times with an approximate likelihood calculation [74], which allows a gamma-Dirichlet prior to describe substitution rates across multiple loci, thereby improving the accuracy of divergence-time estimation [75]. Optimal scheme for partitioning of the molecular clock(s) was tested for using ClockstaR 2.0.1 [76]. The ML phylogenetic tree topology from the 77 concatenated PCGs was used for divergence time estimation, and the ML branch lengths were estimated using the BASEML program in PAML under the GTR substitution model. For the gamma-Dirichlet prior for the overall substitution rate (rgene gamma), we used a quite diffuse (uninformative) prior =1. We used 14 highly reliable fossils from eudicot orders and three Brassicales fossils (Additional file 1: Table S6). All fossils were carefully selected according to their original descriptions and calibrations by past researches. Based on the mean estimate from three codon partitions using the strict molecular clock assuming 136 Ma constraint at the root, an average of the eudicot-monocot split [46], the gamma-Dirichlet prior for the overall substitution rate (rgene gamma) was set at G (4, 80, 1). The gamma-Dirichlet prior for the rate-drift parameter (sigma2 gamma) was set at G (1, 10, 1).

All calibration constraints were not rigorously constrained (specified with 2.5% tail probabilities above or below their limits; this is a built-in function of MCMCTREE). The independent rates model (clock = 2 in MCMCTREE) [77] was used to specify the prior of rates among the internal nodes, which followed a log-normal distribution. The three parameters (birth rate λ, death rate μ, and sampling fraction ρ) in the birth-death process with species sampling were specified as 1, 1, and 0, respectively. After a burn-in period of 1,000,000 cycles, the MCMC run was sampled every 250 cycles until a total of 20,000 samples were collected. Two separate MCMC runs were compared for convergence with two different random seeds and similar results were observed. To explore the influence of our fossil calibrations on age estimates, we conducted four separate analyses testing the inclusion of various fossil combinations (Additional file 1: Table S7).

## Additional files

Additional file 1:
**Table S1.﻿﻿ **Species and data discription of cp genome used in this study. **Table S2.** Comparison of sequence length and GC content among Brassicales chloroplast genomes. **Table S3.** Comparison of fast-, intermediate-, and slow-evolving plastid protein-coding genes. **Table S4.** Heterogeneity and saturation test results from BaCoCa analysis. **Table S5.** Comparison of partitioning strategies. **Table S6.** Species and lineage specific fossil calibrations. **Table S7.** Comparison of mean and 95% HPD age estimates using different fossils. (XLSX 48 kb)
Additional file 2:
**Figure S1. **Topologies of alternative tree hypothesis used in approximately unbiased test. **Figure S2.** Chronogram of Brassicaceae and 75 outgroup taxa inferred using MCMCTree. **Figure S3.** Alignment view of Brassicales rps16 genes in MEGA6. **Figure S4.** Alignment view of Brassicales ycf15 genes in MEGA6. **Figure S5.** A phylogeny from ML analyses of 77 PCGs using 1st an 2nd codon. **Figure S6.** A phylogeny from ML analyses of 77 PCGs using genes from the IR region. **Figure S7.** A phylogeny from ML analyses of 77 PCGs using 3rd codon. **Figure S8.** A phylogeny from ML analyses of 77 PCGs using all three codons. (PDF 625 kb)

## Abbreviations

AU: Approximately unbiased test
BIC: Bayesian information criterion
BP: Bootstrap
Bp: Base pair
GC: Guaninecytosine
IR: Inverted repeat region
LSC: Large single copy region
PCGs: Protein coding genes
SSC: Small single copy region
