# Supplementary material for: Plastome phylogeny and early diversification of Brassicaceae
Source: BMC Genomics. 2017 Feb 16;18:176. doi: 10.1186/s12864-017-3555-3 (PMC5312533; doi:10.1186/s12864-017-3555-3)
Supplement: Additional file 2: — Figure S1. Topologies of alternative tree hypothesis used in approximately unbiased test. Figure S2. Chronogram of Brassicaceae and 75 outgroup taxa inferred using MCMCTree. Figure S3. Alignment view of Brassicales rps16 genes in MEGA6. Figure S4. Alignment view of Brassicales ycf15 genes in MEGA6. Figure S5. A phylogeny from ML analyses of 77 PCGs using 1st an 2nd codon. Figure S6. A phylogeny from ML analyses of 77 PCGs using genes from the IR region. Figure S7. A phylogeny from ML analyses of 77 PCGs using 3rd codon. Figure S8. A phylogeny from ML analyses of 77 PCGs using all three codons. (PDF 625 kb) [file 12864_2017_3555_MOESM2_ESM.pdf]

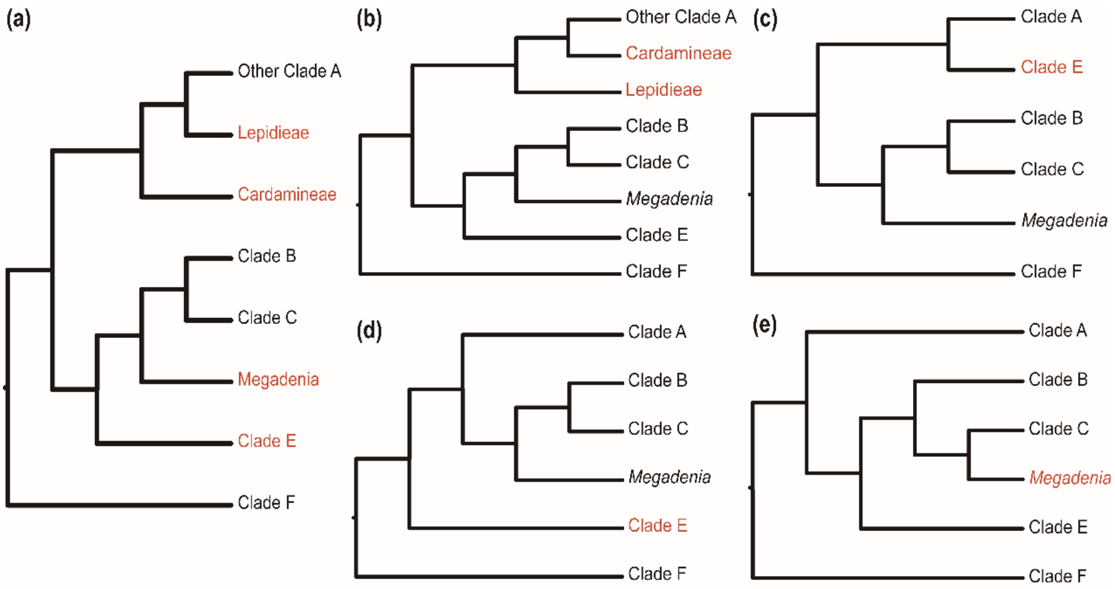

Figure S1. Topologies of alternative tree hypothesis used in approximately unbiased test. Lineages with unstable positions or conflicted with other studies are labelled with red. (a) H1: Phylogeny obtained in this study; (b) H2: Swapping the unstable position of Cardamineae and Lepidieae; (c) H3: Clade E being closer to Clade A, which is derived from ref. 37; (d) H4: Clade E being the outmost of core Brassicaceae (i.e. Clade A-E), which is derived from refs 17 and 34; (e) H5: *Megadenia* (tribe Biscutelleae) being grouped with Clade C, as in ref. 17. Except for the best tree obtain in this study (a), only the lineages of interest in each hypothesis were labelled respectively (b-e).

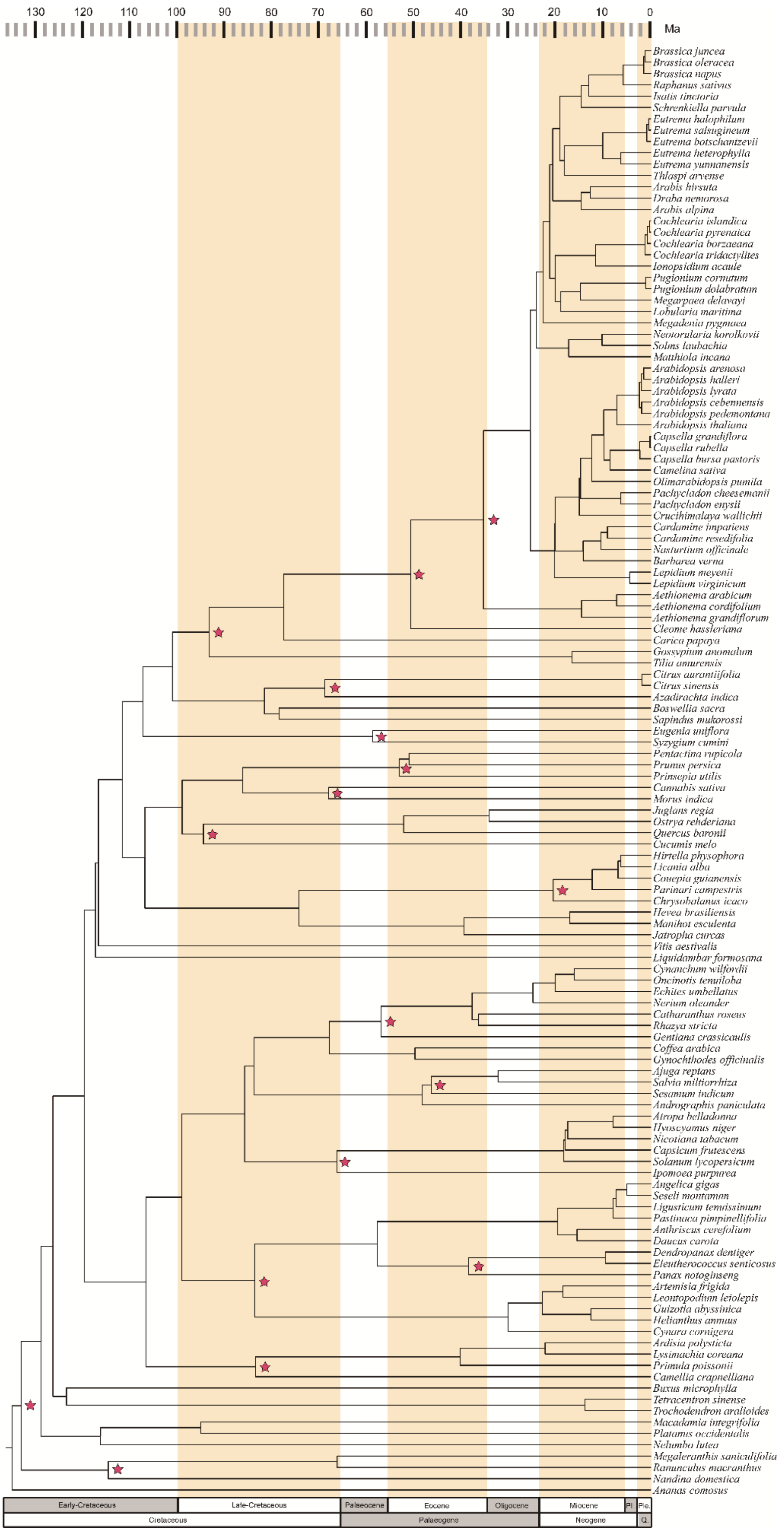

Figure S2. Chronogram of Brassicaceae and 75 outgroup taxa inferred using MCMCTree.

Note: Stars indicate fossil calibrations used in this analysis, see also in Table S6.

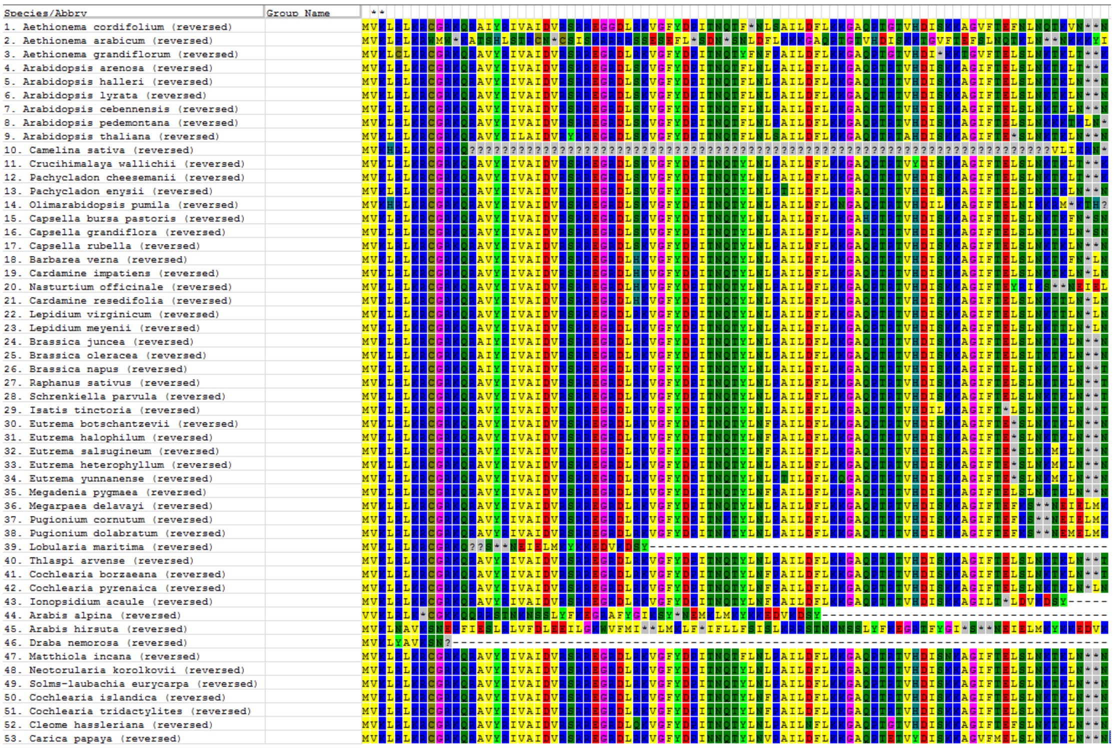

Figure S3. Alignment view of Brassicales *rps16* genes in MEGA6.

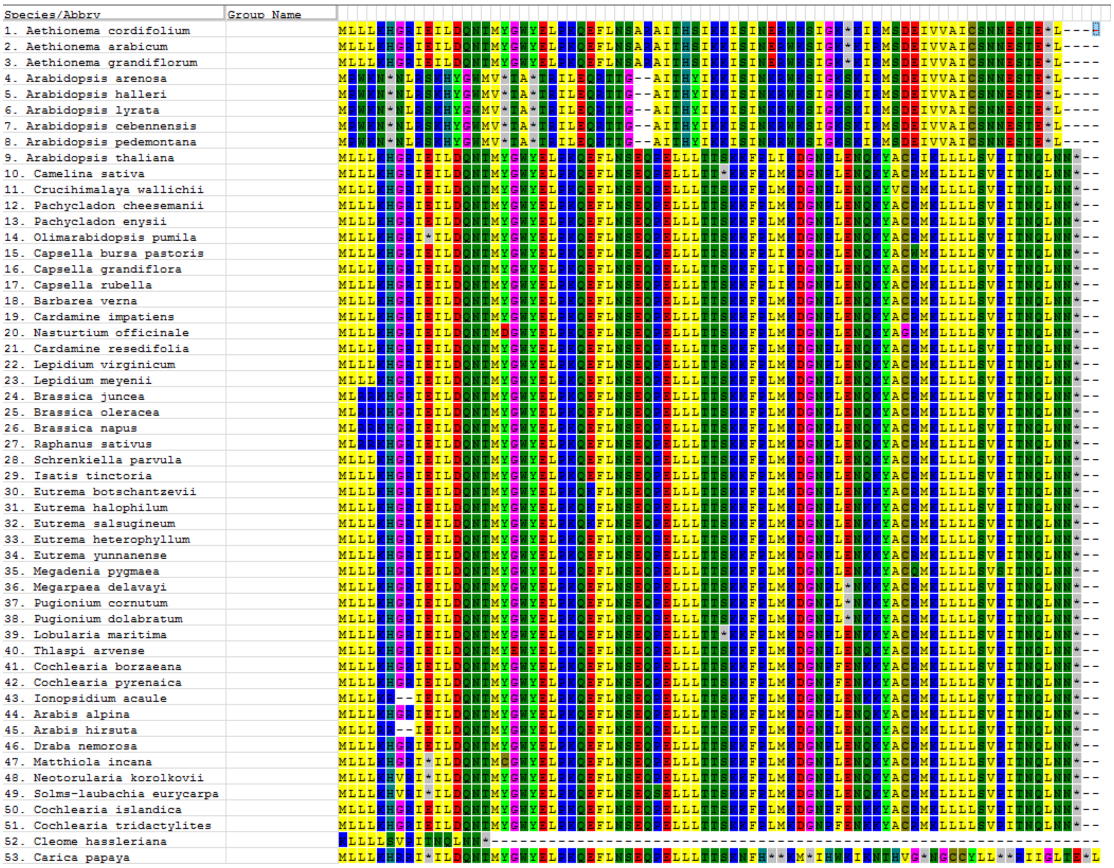

Figure S4. Alignment view of Brassicales *ycf15* genes in MEGA6.

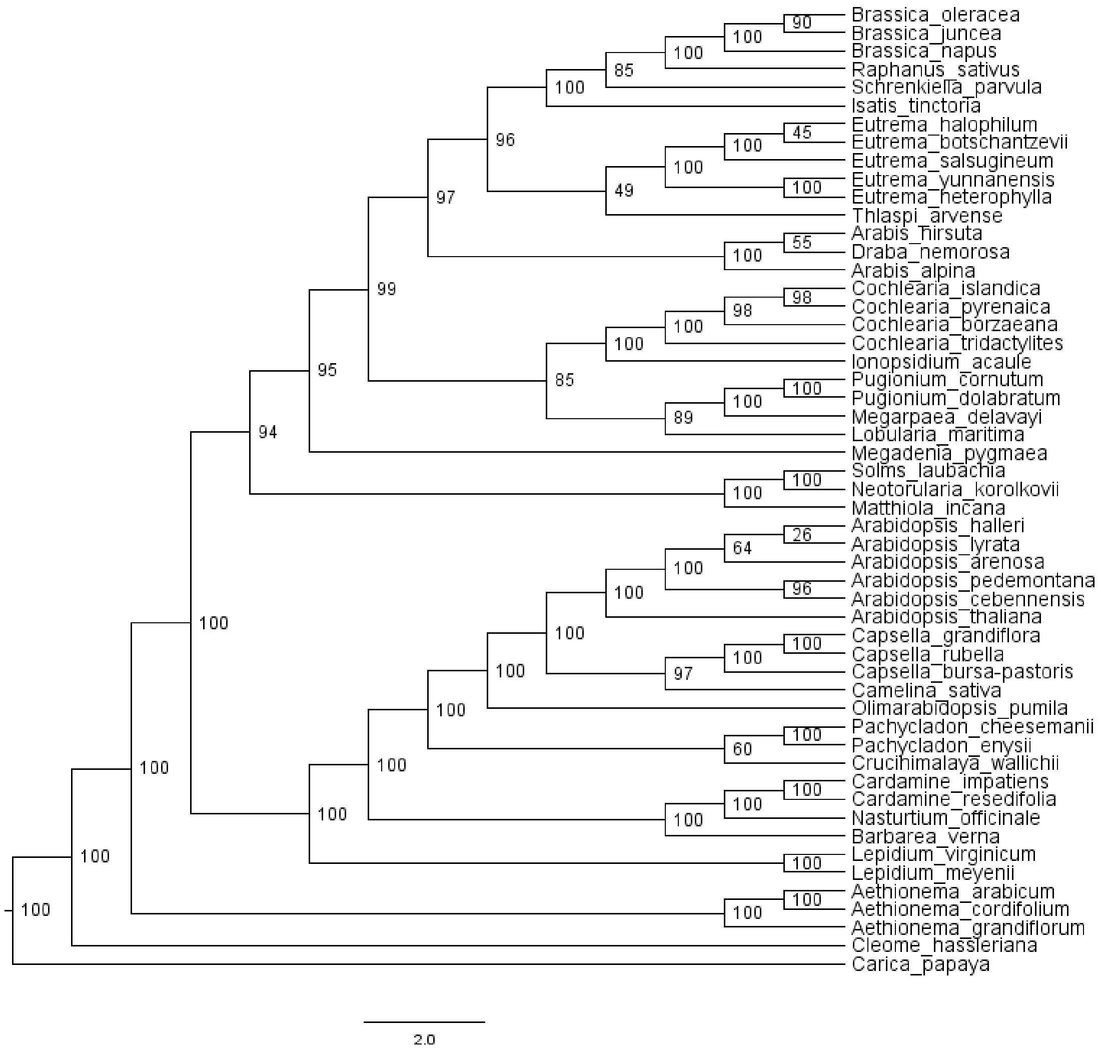

Figure S5. A phylogeny from ML analyses of 77 PCGs using 1st an 2nd codon.

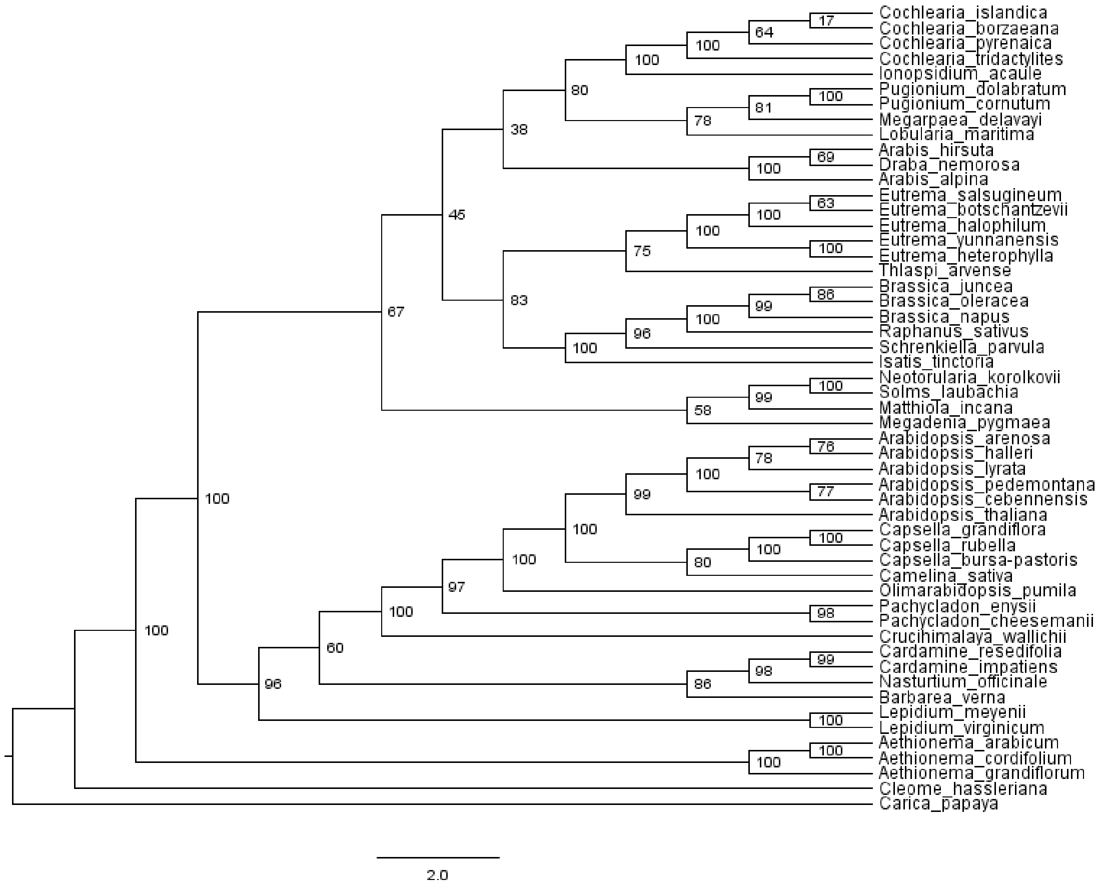

Figure S6. A phylogeny from ML analyses of 77 PCGs using genes from the IR region.

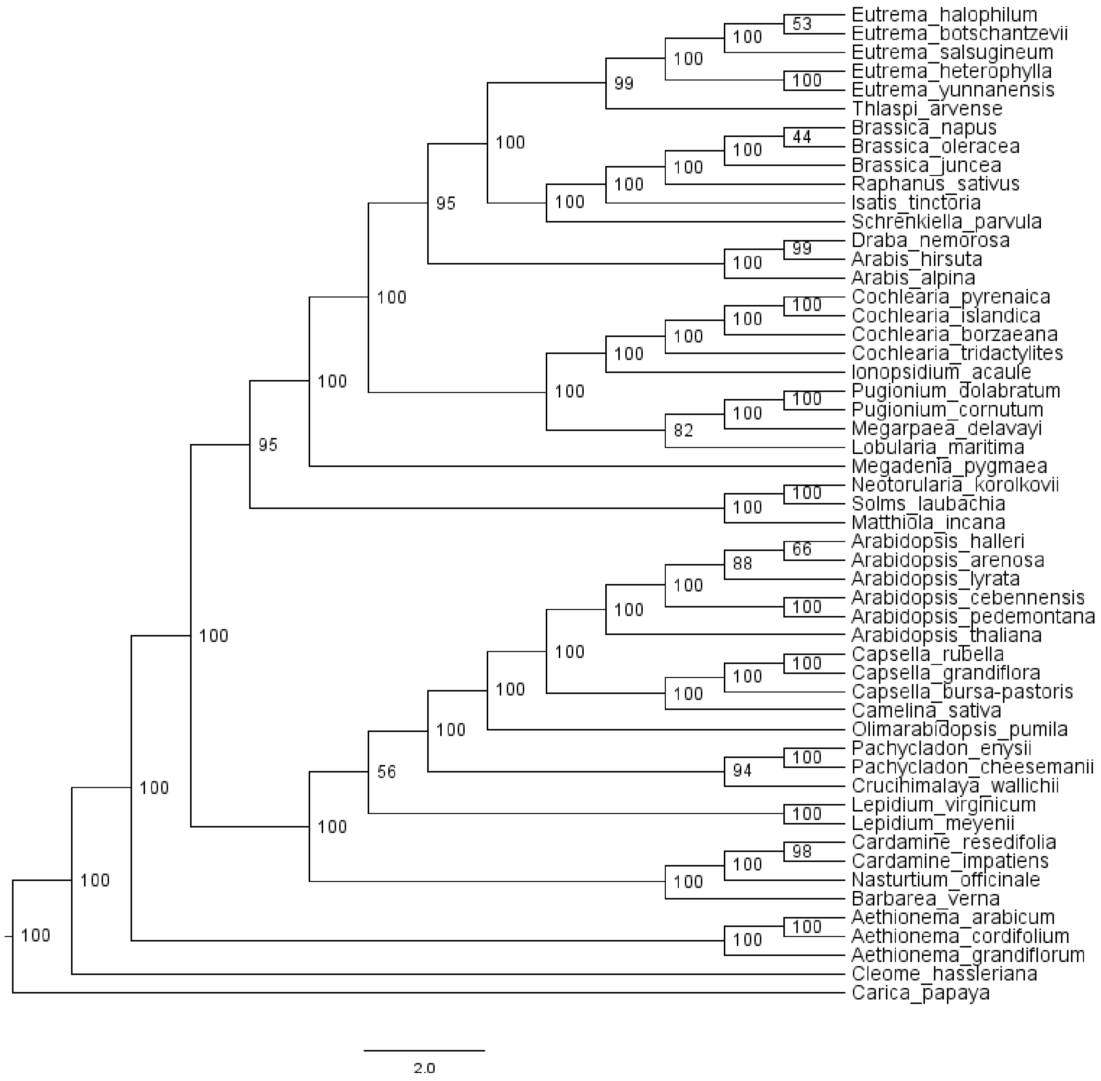

Figure S7. A phylogeny from ML analyses of 77 PCGs using 3rd codon.

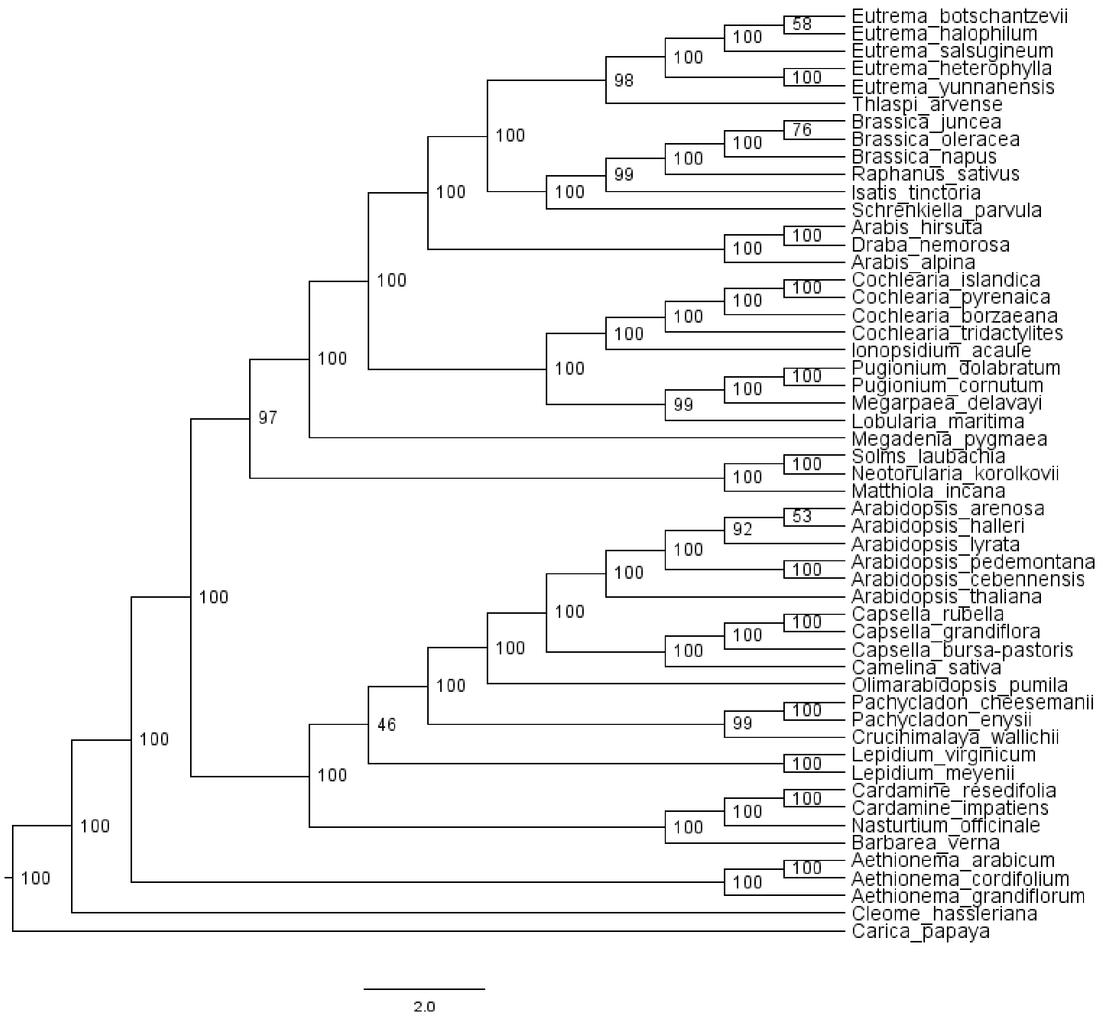

Figure S8. A phylogeny from ML analyses of 77 PCGs using all three codons.
